# The Impact of Host Metabolic Factors on Treatment Outcome in Chronic Hepatitis C

**DOI:** 10.1155/2012/420156

**Published:** 2012-04-22

**Authors:** Savvidou Savvoula, Chrysagis Dimitrios, Papatheodoridis George, Manolakopoulos Spilios, Triantos Christos, Goulis John

**Affiliations:** ^1^4th Department of Internal Medicine, Hippokration General Hospital of Thessaloniki, Medical School of Aristotle University, 54642 Thessaloniki, Greece; ^2^Department of Internal Medicine, Infectious Diseases Hospital of Thessaloniki, Thessaloniki, Greece; ^3^2nd Department of Internal Medicine, Hippokration General Hospital of Athens, University of Athens Medical School, 11527 Athens, Greece; ^4^Division of Gastroenterology, Department of Internal Medicine, University Hospital of Patras, 26504 Rio Patras, Greece

## Abstract

*Background*. Recent data suggest that chronic hepatitis C has to be considered a metabolic disease further to a viral infection. The aim of this study was to elaborate on the complex interactions between hepatitis C virus, host metabolic factors, and treatment response. *Methods*. Demographic, virological, and histological data from 356 consecutive patients were analyzed retrospectively. Hepatic steatosis, obesity, and insulin resistance were examined in relation to their impact on treatment outcome. Comparison between genotype 1 and 3 patients was performed to identify differences in the determinants of hepatic steatosis. *Results*. Histological evidence of hepatic steatosis was found in 113 patients, distributed in 20.3%, 9.0%, and 2.5% for grades I, II, and III, respectively. Hepatic steatosis was associated with past alcohol abuse (*P* = 0.003) and histological evidence of advanced fibrosis (*P* < 0.001). Older age (OR 2.51, *P* = 0.002), genotype (OR 3.28, *P* < 0.001), cirrhosis (OR 4.23, *P* = 0.005), and hepatic steatosis (OR 2.48, *P* = 0.001) were independent predictors for nonresponse. Correlations of hepatic steatosis with alcohol, insulin resistance, and fibrosis stage were found similar for both genotypes 1 and 3. *Conclusions*. Host metabolic factors may predict treatment outcome, and this impact remains significant even in genotype 3, where steatosis has been believed to be exclusively virus related.

## 1. Introduction

Current treatment of chronic hepatitis C (CHC) consists of pegylated interferon alpha (PegIFN*α*) plus ribavirin with an overall sustained virological response (SVR) of 54–63% [1–3]. Even with high adherence to treatment duration, SVR rates remain suboptimal, and several host and viral factors, like age, gender, ethnicity, genotype, and stage of hepatic fibrosis, have been identified as influencing the rate of CHC progression as well as the response to antiviral therapy [4–6].

Hepatic steatosis is a common histological feature of CHC, occurring in approximately 50% [7], twice as often as would be expected in the general population by simple coexistence of nonalcoholic fatty liver disease (NAFLD) and CHC [8, 9]. Even after exclusion of the usual causes of steatosis, such as obesity, diabetes, alcohol, and drugs, the prevalence of steatosis is still around 30–40% [10, 11]. The majority of cases account for mild steatosis, affecting less than 30% of hepatocytes [7].

The pathogenesis of hepatic steatosis in the setting of CHC has been reported to be dual [7, 12, 13]; in the so-called “metabolic” steatosis, fat accumulation in the liver is the result of host metabolic factors like obesity, alcohol consumption, and diabetes, in a way that resembles the derangement of hepatocytes' lipid metabolism in NAFLD [11, 14, 15]. On the other hand, hepatitis C virus (HCV) itself has a direct steatogenic effect [12, 16, 17], as viral structural and nonstructural proteins localize on lipid droplets, interact with apolipoproteins, and interfere with molecular pathways of lipid metabolism [18]. In the setting of this viral-induced steatosis, several reports have demonstrated that exclusively HCV genotype 3 is cytopathic to the liver leading to a more prevalent and a more severe in extent steatosis. In this case, hepatic steatosis has been found to correlate with HCV RNA and to improve after viral clearance [19]. However, several issues concerning this genotype-specific steatosis remain to be elucidated, as experimental models of transgenic mice have used constructs derived from HCV genotype 1 isolates [7, 19, 20], and genomic studies have failed so far to explain the greater propensity of genotype 3 to cause steatosis [21]. Furthermore, several authors have suggested that, as host and viral interactions are far more complex than previously described, more “mixed” types of steatosis would be recognized in the future, especially as obesity tends to affect younger generations [5, 13].

Apart from hepatic steatosis [6, 22–25], researchers have focused on other metabolic factors like obesity [26, 27] and, more recently, insulin resistance (IR) [28–30]. Proposed mechanisms for decreased effectiveness of antiviral therapy include enhanced fibrosis secondary to hepatic steatosis and IR, altered immune responses, and distortion of hepatocyte binding for interferon secondary to hepatic fat deposition [14].

The aim of the present study is to provide a clearer understanding about the interaction between hepatic steatosis, obesity, IR, and current antiviral therapy. Furthermore, this study examines possible virus-related differences concerning the impact of the metabolic profile of the host on treatment outcome.

## 2. Patients and Methods

### 2.1. Patient Selection

**Figure 1:**
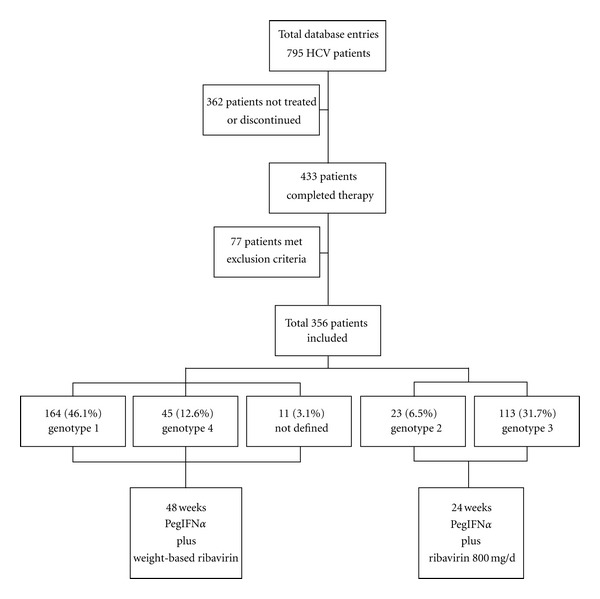
Patients entering the study.

Databases from five major hepatology units were used to select data from CHC patients who had been subjected to pretreatment liver biopsy. These databases provided demographic, serological, virological, and histological data from a total of 795 consecutive patients. Patients, who neither received nor completed therapy, were excluded from the study. According to inclusion criteria, patients had to be naïve, aged above 18 years, and have received therapy for more than 80% of the recommended treatment duration.  Figure 1 demonstrates patients entering the study, as well as distribution of genotypes, and type of antiviral therapy.

### 2.2. Demographic Data

Databases were used to determine patients' age, gender, history of alcohol consumption, and somatometric measurements. Previous intravenous drug use (IVDU) was also recorded as a possible mode of HCV transmission. Past alcohol abuse was defined as a consumption of more than 120 g alcohol per week, at least 6 months prior to the beginning of treatment. Weight and height measurements were used to calculate body mass index (BMI).

### 2.3. Laboratory Investigations

Baseline serum alanine (ALT) and aspartate aminotransferases (AST) were measured by standard biochemical analysers. Abnormal values were considered as values just above the upper limit of normal.

In a subgroup of CHC patients, an overnight fasting blood sample was taken in advance of therapy onset for further assessment of IR using the homeostasis model assessment (HOMA), as well as measurement of total cholesterol, serum triglyceride concentration, fasting glucose, insulin, and C-peptide. Patients with HOMA ≥ 2 were considered to be insulin resistant [28].

### 2.4. Virology Assessments

All patients were HCV-RNA positive by qualitative polymerase chain reaction (PCR). HCV RNA was determined by reverse transcriptase PCR using commercial kits (Amplicor HCV, Roche Diagnostics, Branchburg, NJ). HCV genotyping was performed with a second-generation reverse hybridization line probe assay (INNO-LiPA HCV II, Belgium). Baseline high viral load was defined as HCV RNA greater than 800,000 IU/mL.

### 2.5. Liver Histology

Inflammatory activity and fibrosis were assessed according to the METAVIR scoring system [31] (4 stages for activity: A0–A3 and 5 stages for fibrosis: F0–F4) or the modified Ishak score [32] (Histology Activity Index (HAI) scale 0–18 and fibrosis scale 0–6). Severe inflammation was considered as having either A3 or HAI > 12. Advanced fibrosis was defined as stage ≥F3 or ≥4, while cirrhosis was defined as having stage F4 or stages ≥5, for the two scoring systems, respectively.

Steatosis was semiquantified by determining the proportion of hepatocytes containing fat droplets. According to Brunt's classification [33], specimens were assigned a grade (0 to III) based upon the percentage of affected hepatocytes. Grade 0 was considered as absence of hepatic steatosis.

### 2.6. Treatment Outcomes

All patients included in the study were treated with combination therapy of either PegINF*α*-2a (180 *μ*g/w) or PegIFN*α*-2b (1.5 *μ*g/kg/w), combined with ribavirin, according to guidelines [34], as shown in Figure 1. Adherence to standard dosage was defined as positive when patients constantly received more than 80% of the recommended dosage. Primary endpoint of the study was SVR, defined as undetectable HCV RNA 24 weeks after the end of treatment.

### 2.7. Statistical Analysis

Treatment outcome was analyzed as the dependent dichotomous categorical variable. Statistical tests of chi-square, Student's *t*-test or Mann-Whitney, were used as appropriate for group comparisons. Finally, a multiple logistic regression analysis model was applied in order to determine possible independent prognostic factors of SVR. All statistical analyses were made using SPSS v11.5. *P* values were considered statistically significant at the 0.05 level.

## 3. Results

### 3.1. Baseline Host and Viral Characteristics

A total of 356 consecutive CHC patients were included in the study. Patient baseline characteristics were 197 (55.3%) male, 203 (57.8%) aged above 40, 111 (31.3%) former IVDUs, 41 (12%) past alcohol abusers, and 49 (36.6%) overweight with mean BMI 23.3 ± 4.0 kg/m^2^. The majority of HCV patients (96.2% and 77.2%) had abnormal baseline ALT and AST, respectively, while approximately half of them (*n* = 67, 42.7%) presented with high viral load. Liver histology found 56 patients (15.9%) with advanced fibrosis, 21 (6%) with cirrhotics, 17 (5.1%) with severe necroinflammatory activity, and 113 (31.7%) with evidence of hepatic steatosis. Demographic, virological, and histological data according to the genotype are shown in Table 1.

### 3.2. Treatment Outcome

A total of 251 (70.5%) patients achieved SVR, while the remaining 105 patients did not respond or had a viral relapse. SVR rates were 67.7%, 73.9%, 86.7%, and 40.0% for genotypes 1, 2, 3, and 4, respectively.

Variables with strong correlation to treatment outcome were selected for statistical analysis with the multiple logistic regression method (Table 2). IVDU was strongly correlated with genotype 3 (*χ* ^2^, *P* ≤ 0,001) and, thus, was excluded from the model. Four categorical variables with strong correlation to treatment outcome were selected for the multiple logistic regression analysis: presence of cirrhosis, genotype 1 or 4 compared to genotypes 2 or 3, age above 40 years, and hepatic steatosis. The results of the multiple logistic regression analysis including the odd ratios and 95% confidence intervals (CI) are shown in Table 3. Model validity was tested with the Hosmer-Lemeshow's goodness-of-fit test (*P* = 0.969 > 0.05).

### 3.3. Impact of Hepatic Steatosis on Treatment Outcome

A total of 241 patients (67.7%) had no steatosis in liver biopsy, while 72 (20.2%), 32 (9.0%), and 9 (2.5%) patients had histological evidence of grade I, II, and III hepatic steatosis, respectively.

Presence of hepatic steatosis was found to be associated with past alcohol abuse (*P* = 0.003), high viral load (*P* = 0.008), abnormal ALT and AST (*P* = 0.015 and *P* = 0.026 resp.), severe necro-inflammatory activity (*P* < 0.001), and advanced fibrosis (*P* < 0.001). As shown in Table 4, genotype distribution did not differ between two groups. However, the majority of patients with grade III hepatic steatosis in liver biopsy (6 out of 9, 66.6%) were patients infected with HCV genotype 3 (data not shown).

SVR was achieved in 185 patients without hepatic steatosis (76.8%) versus 64 patients (56.6%) with steatosis ≥33% in pretreatment liver biopsy (*P* < 0.001). As already mentioned, presence of hepatic steatosis was found to be a strong predictor for non-response, independently of age, genotype, and fibrosis stage.

### 3.4. Impact of BMI on Treatment Outcome

Data about BMI measurements were available only for 134 patients (37.6% of total). BMI ranged between 14.4 and 34.1 kg/m^2^, with a mean value 23.7 ± 4.1 kg/m^2^. The majority of patients (63.4%) had a BMI within normal, while 29.1% were overweight and 7.5% were obese.

BMI was found to be higher in nonresponders (24.6 ± 4.5 versus 23.1 ± 3.8 kg/m^2^, Student's *t*-test, *P* = 0.035), in older patients (*P* = 0.028), and in males (*P* = 0.024) but not in former alcoholics (*P* = 0.059) or cirrhotics (*P* = 0.09). No statistical significant associations were found regarding hepatic steatosis. However, taking into account the strong association of steatosis with alcohol and fibrosis, when alcohol abusers and cirrhotics were excluded, the percentage of overweight patients increased in higher grades of hepatic steatosis (27.8%, 30.3%, 35.7%, and 50% for grades 0, I, II, and III, resp.) (data not shown).

### 3.5. Impact of IR on Treatment Outcome (Subgroup Analysis)

HOMA was estimated in a small subgroup of 78 patients, all noncirrhotic. This subgroup was representative of the total, as no statistical significant differences in patient and viral baseline characteristics were recorded. Compared to the total patient sample, this subgroup of patients presented higher prevalence of hepatic steatosis (59.1% versus 31.7%).

Baseline patient metabolic status, included measurements of fasting glucose levels (median 96 mg/dL, 25th–75th interquartile range (IqR): 90–102), fasting insulin (median 9 mIU/mL, IqR: 5.0–14.5), c-peptide (median 2.3 ng/mL, IqR: 1.6–3.0), total cholesterol (median 172 mg/dL, IqR: 140–202), and serum triglycerides (median 83 mg/dL, IqR: 69–108). Median HOMA was 2.2 (IqR: 1.1–4.1). Half of these patients (51.1%) were found insulin resistant by definition (HOMA ≥2).

**Figure 2:**
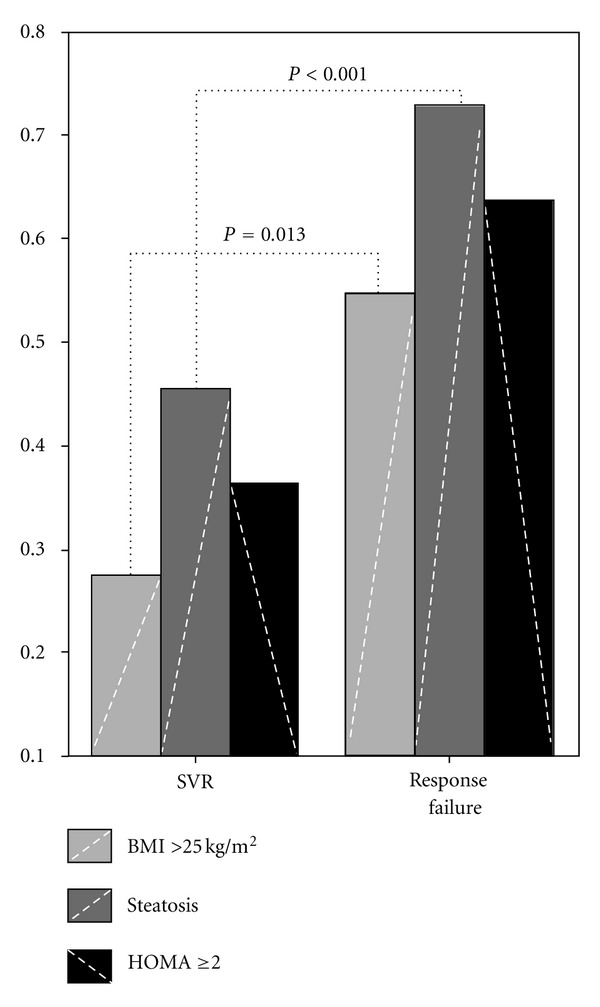
Prevalence of host metabolic factors in responders and nonresponders.

IR was associated with older age (*χ* ^2^, *P* = 0.041), BMI (Student's *t*-test, *P* < 0.001) and presence of hepatic steatosis (*χ* ^2^, *P* < 0.001). HOMA correlated with BMI after logarithmic transformation (Pearson's correlation, *r* = 0.544, *P* = 0.006) and with presence of hepatic steatosis (Mann-Whitney test, *P* < 0.001). Finally, prevalence of insulin resistance was greater in non-responders than responders (63.6% versus 38.5%), but this difference did not reach the level of statistical significance (Figure 2).

### 3.6. Impact of HCV Genotype on Host Metabolic Profile

Only genotype 1 (*n* = 164) and genotype 3 (*n* = 113) HCV patients were selected for further statistical analysis, in order to examine possible differences in their metabolic profile. Genotype 3 patients were mostly younger (*P* < 0.001), former IVDUs (*P* < 0.001), and responded better to antiviral treatment (86.7% versus 67.7%, *P* < 0.001). Differences in hepatic steatosis were also recorded (*χ* ^2^ test, *df* = 3, *P* = 0.032), while BMI was similar (23.6 ± 4.8 versus 23.1 ± 3.4 kg/m^2^, Student's *t*-test, *P* = 0.561), and differences in HOMA did not reach the level of statistical significance (median HOMA 2.7 (IqR: 1.5–4.2) in genotype 1 versus 1.8 (IqR: 0.8–3.1) in genotype 3, and Mann-Whitney test, *P* = 0.315).

Further statistical analysis, as shown in Table 5, revealed the following.

- Genotype 3 HCV patients were mostly younger, former IVDUs, but this did not seem to be related to the presence of steatosis.
- Gender and BMI did not differ between genotypes 1 and 3 nor influenced the presence of hepatic steatosis.
- Alcohol abuse was strongly associated with presence of hepatic steatosis independently of genotype.
- HOMA was higher in patients with hepatic steatosis independently of genotype.
- High viral load influenced the presence of hepatic steatosis only in genotype 3 patients.
- Severe necro-inflammatory activity was associated with hepatic steatosis in genotype 1 patients but not in genotype 3 patients.
- Strong correlations of hepatic steatosis and fibrosis were recorded in both genotypes.

## 4. Discussion

### 4.1. Treatment Outcome and Impact of Host Metabolic Factors

In this cohort of patients, SVR rates ranged between 40 and 86.7% according to genotype, clearly higher than those previously reported in large clinical trials [1–3]. This could partly be explained by the fact that this was a retrospective study with strict defined exclusion criteria and no intention to treat analysis. Comparison between different genotypes revealed that young age was the strongest determinant for the high SVR rate encountered in genotype 3 patients. On the other hand, genotype 4 presented the worst prognosis. Systematic review of the literature showed that data on the efficacy of current antiviral therapy of genotype 4 CHC infection are limited and contradictory [35]; SVR rates of 60% reported in endemic areas [35, 36] are at least 2-times higher than those encountered in Southern Europe [37, 38]. In Greece, genotype 4 accounts for approximately 15% of all HCV infections and is generally considered as “difficult to treat” in everyday clinical practice [39]. Certainly, this contradiction has to be addressed in future studies.

The results of this study indicate that histological evidence of hepatic steatosis in pretreatment liver biopsy is an independent prognostic factor for nonresponse to current antiviral therapy. BMI and HOMA were also associated with treatment outcome. However, their impact could not have been established in the multivariate logistic regression, probably because of the strong associations between hepatic steatosis, BMI, and IR.

Several reports in the literature have already documented the importance of host metabolic factors on treatment response, defining either steatosis [22, 23] or IR [28, 29] as independent factors. Systematic review of the literature reveals that there is not a certain metabolic factor to determine treatment response, but a total metabolic burden of the host that interferes with the therapeutic process and decreases the possibility of achieving SVR.

### 4.2. Determinants of Hepatic Steatosis

The overall prevalence of hepatic steatosis in our study was 31.8%, distributed in 20.8%, 9%, and 2.5% for grades I, II, and III, respectively, similar to those reported previously [6, 23, 40]. The reported prevalence of hepatic steatosis in CHC patients ranges between 34.8 and 81.2% [7]. This wide range itself indicates that several independent factors may influence presence of steatosis.

One major determinant of steatosis, supported by most authors, is genotype 3 [6, 22, 40–43], which directly leads to hepatocyte steatogenesis. In this setting, association of steatosis with HCV RNA mirrors this direct cytopathic effect of HCV, and also explains why genotype 3 CHC presents with more severe grades of hepatic steatosis [38]. Correlation of fatty liver and increased BMI has been found in some studies [6, 23, 24, 41–43], even though this correlation has been suggested to be limited to nongenotype 3 CHC patients [7, 43, 44]. The association of hepatic steatosis and advanced fibrosis, supported by the “two hit” theory, is not a constant finding [6, 22, 36–38]. In our study, associations of hepatic steatosis with fibrosis were recorded in both genotypes, while with necro-inflammatory activity were recorded only in genotype 1 patients, which implies possible different interactions concerning mechanisms of hepatic steatogenesis. It is also still not clear whether steatosis correlates with age and aminotrasferase activity or whether these variables act as confounding factors.

Finally, it has already been well documented that both alcohol and HCV induce liver steatosis by acting synergistically in the hepatocyte. Toxic effects of ethanol and its metabolites include mainly alterations on mitochondrial lipid oxidation and proinflammatory cytokine production. Using the relatively small cutoff limit of 120 g alcohol per week in our study, we found that past alcohol intake was associated with presence of hepatic steatosis irrespective of genotype. However, it is still debated in the literature whether past alcohol consumption, and in what extent, could influence viral steatogenic mechanisms.

### 4.3. Genotype-Related Differences

Results derived from the comparison between genotype 1 and genotype 3 HCV patients confirmed the steatogenic and cytopathic effect of genotype 3, as prevalence of hepatic steatosis was found higher in the latter. Viral load was also found to be associated with hepatic steatosis only in genotype 3 patients, similar to other reports [6, 41].

Furthermore, in a similar way with the study of Fartoux and colleagues [44], hepatic steatosis was found to be associated with past alcohol intake, insulin resistance, and presence of advanced fibrosis. Inflammation, although mostly mild, as expected, and not different between two genotypes, was found to be associated with hepatic steatosis only in genotype 1 patients. This could possibly be an indication that viral-related steatosis encountered in genotype 3 results less from noninflammatory processes in liver parenchyma.

Another interesting difference with the study of Fartoux was the fact that neither BMI nor HOMA was found to be different between two genotypes, indicating that metabolic factors are present even in genotype 3 HCV patients, where steatosis was believed to be exclusively virus related.

## 5. Conclusion

The close association of HCV and hepatic steatosis has already been well documented by several investigators. Recent data also suggest that CHC has to be considered a metabolic disease further to a viral infection. Our study, despite the limitations by its retrospective nature, indicates that metabolic factors may impact treatment outcome of standard antiviral therapy, and that hepatic steatosis in genotype 3 has to be considered as the result of complex interplay between metabolic and viral factors and not exclusively virus related as previously reported. This “mixed type” of hepatic steatosis is expected to be increasingly recognized in the future. In this setting, and in the era of new emerging antivirals, a metabolic approach could be helpful, especially for those patients who do not benefit from current antiviral treatment.

## Figures and Tables

**Table 1:** Demographic, virological, and histological data according to genotype.

	Genotype 1 *n* = 164	Genotype 2 *n* = 23	Genotype 3 *n* = 113	Genotype 4 *n* = 46	*P* values*
Gender (males%)	87 (53.0)	12 (52.2)	66 (58.4)	24 (53.3)	0.826
Age ≥ 40 years (*n*%)	110 (67.5)	19 (82.6)	41 (36.9)	30 (68.2)	<0.001
IVDU (*n*%)	40 (24.4)	3 (13.0)	59 (52.7)	6 (13.3)	<0.001
Alcohol abuse (*n*%)	15 (9.3)	2 (8.7)	17 (15.7)	6 (14.0)	0.392
BMI (kg/m^2^)	23.6 ± 4.8	26.4 ± 3.9	23.2 ± 3.4	23.5 ± 4.1	0.144
Overweight (*n*%)	15 (32.6)	7 (70.0)	18 (32.1)	8 (40.0)	0.126
High viral load (*n*%)	23 (33.3)	3 (37.5)	32 (55.2)	9 (42.9)	0.101
Hepatic steatosis (*n*%)	46 (28.2)	8 (34.8)	37 (32.7)	16 (35.6)	0.721
Severe inflammation (*n*%)	10 (6.5)	0 (0)	3 (2.9)	2 (4.7)	0.406
Advanced fibrosis (*n*%)	23 (14.1)	4 (17.4)	17 (15.3)	11 (24.4)	0.411
Cirrhosis (*n*%)	7 (4.3)	1 (4.3)	8 (7.2)	5 (11.1)	0.356

*Comparison between genotypes with *χ* ^2^ (nonparametrical) or one-way ANOVA (parametrical variables).

**Table 2:** Variable associations with SVR.

	SVR (*n* = 251)	Response failure (*n* = 105)	*P* values
Gender (males%)	145 (57.8)	52 (49.5)	0.154
Age ≥ 40 years (*n*%)	123 (49.60)	80 (77.7)	**<0.001**
IVDU (*n*%)	95 (37.8)	16 (15.4)	**<0.001**
Alcohol abuse (*n*%)	27 (11.2)	14 (14.1)	0.442
BMI (kg/m^2^)	23.1 ± 3.8	24.6 ± 4.5	** 0.035**
Overweight (*n*%)	24 (28.6)	25 (50.0)	** 0.013**
Genotype distribution			**<0.001**
Genotype 1	111 (45.5)	53 (52.5)	
Genotype 2	17 (7.0)	6 (5.9)	
Genotype 3	98 (40.2)	15 (14.9)	
Genotype 4	18 (7.4)	27 (26.7)	
High viral load (*n*%)	39 (40.6)	28 (45.8)	0.515
Abnormal ALT (*n*%)	236 (96.7)	95 (95.0)	0.447
Abnormal AST (*n*%)	199 (86.1)	77 (86.5)	0.931
Hepatic steatosis (*n*%)	64 (25.7)	49 (46.7)	**<0.001**
Severe inflammation (*n*%)	9 (3.8)	8 (8.5)	0.080
Advanced fibrosis (*n*%)	26 (10,5)	30 (28,6)	**<0.001**
Cirrhosis (*n*%)	7 (2,8)	14 (13,3)	**<0.001**
Type of IFN			0.987
PegIFN*α*-2a (*n*%)	69 (28.6)	27 (28.7)	
PegIFN*α*-2b (*n*%)	172 (71.4)	67 (71.3)	
Dosage adherence (*n*%)	173 (80.8)	60 (82.2)	0.799

**Table 3:** Multiple logistic regression analysis for nonresponse.

Variables	B	SE	Wald	df	*P* values	OR	95% CI	
							Lower	Upper
Age ≥ 40 years	0.92	0.30	9.68	1	0.002	2.5	1.41	4.47
Genotype 1or 4	1.19	0.31	15.05	1	0.0001	3.28	1.80	5.60
Hepatic steatosis	0.91	0.28	10.84	1	0.001	2.48	1.44	4.26
Cirrhosis	1.44	0.52	7.80	1	0.005	4.23	1.54	11.64
Constant	−2.70	0.34	63.15	1	0.0001	0.067		

**Table 4:** Variable associations with presence of hepatic steatosis.

	No steatosis (*n* = 241)	Hepatic steatosis (*n* = 113)	*P* values
Gender (males%)	136 (56.4)	59 (52.2)	0.457
Age ≥ 40 years (*n*%)	132 (55.0)	71 (65.1)	0.075
IVDU (*n*%)	80 (33.3)	31 (27.4)	0.265
Alcohol abuse (*n*%)	20 (8.5)	21 (20.0)	**0.003**
BMI (kg/m^2^)	23.6 ± 3.5	23.6 ± 4.8	0.988
Overweight (*n*%)	18 (34.6)	30 (37.5)	0.736
Genotype distribution			0.721
Genotype 1	117 (49.4)	46 (43.0)	
Genotype 2	15 (6.3)	8 (7.5)	
Genotype 3	76 (32.1)	37 (34.6)	
Genotype 4	29 (12.2)	16 (15.0)	
High viral load (*n*%)	27 (32.9)	40 (54.1)	** 0.008**
Abnormal ALT (*n*%)	231 (97.9)	98 (92.5)	** 0.015**
Abnormal AST (*n*%)	202 (89.0)	74 (79.6)	** 0.026**
Severe inflammation (*n*%)	5 (2.2)	12 (12.0)	**<0.001**
Advanced fibrosis (*n*%)	22 (9.2)	34 (30.1)	**<0.001**
Cirrhosis (*n*%)	9 (3.8)	12 (10.6)	** 0.011**
SVR (*n*%)	185 (76.8)	64 (56.6)	**<0.001**

**Table 5:** Differences of genotypes 1 and 3 in determinants of hepatic steatosis.

	Genotype 1			Genotype 3			*P* values*
	No steatosis *n* = 117	Steatosis *n* = 46	*P* values	No steatosis *n* = 76	Steatosis *n* = 37	*P* values	
Gender (males%)	65 (55.6)	21 (45.7)	NS	42 (55.3)	24 (64.9)	NS	NS
Age ≥ 40 years (*n*%)	75 (64.1)	35 (77.8)	NS	26 (34.7)	15 (41.7)	NS	**0.001**
IVDU (*n*%)	31 (26.5)	9 (19.6)	NS	40 (53.3)	19 (51.4)	NS	**0.005**
Alcohol abuse (*n*%)	7 (6.0)	8 (18.2)	**0.030**	7 (9.7)	10 (27.8)	**0.024**	NS
BMI (kg/m^2^)	24.0 ± 4.2	23.5 ± 5.3	NS	22.9 ± 3.2	23.3 ± 3.5	NS	NS
HOMA	1.6 (1.4–1.6)	4.0 (3.5–4.3)	**0.024**	0.8 (0.7–1.1)	2.8 (2.4–4.1)	**0.016**	NS
High viral load (*n*%)	10 (27.0)	13 (41.9)	NS	13 (40.6)	19 (73.1)	**0.018**	**0.031**
Severe inflammation (*n*%)	2 (1.8)	8 (20.0)	**>0.001**	1 (1.4)	2 (6.3)	0.231	NS
Advanced fibrosis (*n*%)	11 (9.4)	12 (26.1)	**0.011**	5 (6.8)	12 (32.4)	**0.001**	NS
SVR (*n*%)	87 (74.4)	23 (50.0)	**0.005**	70 (92.1)	28 (75.7)	**0.035**	**0.023**

*Comparison between genotype 1 patients with steatosis (*n* = 46) and genotype 3 patients with steatosis (*n* = 37) (columns 3, 6, and 8).

## Abbreviations

ALT: Alanine aminotransferase
AST: Aspartate aminotransferase
BMI: Body mass index
CHC: Chronic hepatitis C
CI: Confidence interval
HAI: Histology Activity Index
HCV: Hepatitis C virus
HOMA: Homeostasis model assessment
IqR: 25th–75th interquartile range
IR: Insulin resistance
IVDU: Intravenous drug use
NAFLD: Nonalcoholic fatty liver disease
PCR: Polymerase chain reaction
PegIFN*α*: Pegylated interferon alpha
SVR: Sustained virological response.
